# 28 Risk Factors and Outcomes of Opioid Dependence After Burn Injury: A Single Center Study

**DOI:** 10.1093/jbcr/irac012.031

**Published:** 2022-03-23

**Authors:** Elliot Walters, Steven E Wolf

**Affiliations:** University of Texas Medical Branch, Dickinson, Texas; University of Texas Medical Branch at Galveston, Galveston, Texas

## Abstract

**Introduction:**

Patients with severe burns often have a prolonged recovery course and frequently opioid pain medications. Several studies showed patients who receive frequent and high doses of opioid medications are at elevated risk of developing opioid dependence. Risk factors for opioid dependence have been established in several fields, including in trauma patients, however opioid dependence within the burn population has not been well studied. In this study we identify risk factors and outcomes for burned patients with opioid dependence.

**Methods:**

We performed a review of a deidentified database that covers our institution comprising over 1.9 million patients. ICD-10 codes were used to identify those with burns. We identified 9150 patients who received treatment for a burn injury between January 1, 2010 and December 31, 2020. From this cohort 130 patients (1.4%) developed documented opioid dependence. Patients from each cohort were balanced by propensity score matching. The database was then examined to determine treatment type and concomitant diagnoses.

**Results:**

Prior to matching we found a significant increase in mortality, chronic pain, non-opioid substance abuse, depression, and use of opioid and non-opioid medication (p< 0.05) for those with opioid dependence. After propensity score matching, we found no significant increase in mortality or depression (p >0.05). Chronic pain and non-opioid substance abuse remained elevated (OR 2.7, CI 1.6, 4.4; OR 2.4, CI 1.3, 4.5, p< 0.05, respectively). Those who developed opioid dependence were more likely to receive opioid and non-opioid pain medication (p< 0.05), but these were not more likely to receive IV opioid pain medication (p >0.05). However, they were more likely to receive IV opioid pain medication more frequently (p< 0.05). Interestingly, patients who developed opioid dependence were more likely to follow up post-operatively and to receive anti-depressant and anti-epileptic (gabapentin and pregabalin) medications (p< 0.05).

**Conclusions:**

Here we presented data on patients who developed opioid dependence following burn injury. These patients appear to receive more pain medication and receive it more frequently. We did not find a correlation of opioid dependence to depression or patient compliance. Characterizing the patient who develops opioid dependence will better help clinicians to identify patients at risk and direct their care accordingly. Further investigation is indicated to determine the impact these factors have and how these might be mitigated.

